# Exploring pathways to develop interprofessional identity: a moderated mediation study

**DOI:** 10.1007/s10459-025-10441-8

**Published:** 2025-05-15

**Authors:** Qing He, John Ian Wilzon T. Dizon, George L. Tipoe, Xiaoai Shen, Fraide A. Ganotice

**Affiliations:** Bau Institute of Medical and Health Sciences Education, Li Ka Shing Faculty of Medicine, The University of Hong Kong, Hong Kong SAR, China

**Keywords:** Interprofessional identity, Interprofessional education, Professional self-efficacy, Motivational beliefs, Health professions students

## Abstract

Interprofessional identity (IPI) is crucial in shaping the perceptions, knowledge, and collaborative skills of health professions learners. Its development is influenced by external factors (educational and practice contexts) and internal factors (motivational beliefs and confidence). Despite its importance, a comprehensive understanding of IPI formation is limited. This study proposed a theoretical model to clarify how professional self-efficacy and motivational beliefs contribute to IPI development, moderated by learning gains and satisfaction within interprofessional education (IPE). A longitudinal study with 473 health professions students was conducted during a 3-week IPE simulation course. Data were collected at the start and end of an IPE simulation course through questionnaires assessing professional self-efficacy, motivational beliefs, IPI, and program learning gains and satisfaction at two time points in 2023. A bootstrapped moderated mediation analysis using PROCESS Macro was employed to examine the mediating role of motivational beliefs between professional self-efficacy and IPI. Additionally, the study explored whether learning gains and satisfaction moderated the link between motivational beliefs and IPI. Professional self-efficacy significantly predicted IPI, with motivational beliefs mediating this relationship. The mediation effect was strengthened by students’ perceived learning gains and satisfaction with the IPE program, indicating a significant index of moderated mediation (B = 0.073, BootSE = 0.026, 95% BootCI [0.020, 0.120]). Effective IPE initiatives should enhance professional self-efficacy and motivational beliefs to develop IPI among health professions students. Creating a supportive interprofessional learning environment is essential, fostering engagement and satisfaction to build a resilient IPI, ultimately enhancing collaborative practices and patient care quality.

**Supplementary Information:**

The online version contains supplementary material available at 10.1007/s10459-025-10441-8.

## Introduction

Interprofessional collaboration is crucial for improving patient safety, providing effective patient care, and mitigating medication errors (Alqenae et al., 2020; Reeves et al., 2013). In interprofessional teams, healthcare professionals learn about and appreciate the roles of other disciplines, fostering trust and collaborative decision-making that extend beyond individual expertise (D’amour & Oandasan, 2005). To achieve effective collaboration, interprofessional education (IPE) programs have been intentionally developed for health professions students, aiming to break down silos, stereotypes, and hierarchies while building interprofessional competencies (Reeves et al., 2013). Notably, a key focus of IPE is to cultivate an understanding of and proficiency in navigating roles within interprofessional teams, leading to the development of interprofessional identity (IPI; Burford, 2012; McGuire et al., 2020; Reinders & Krijnen, 2023).

Professional identity, understood as the internalization of a profession’s norms, values, and roles that shape how individuals perceive and enact their responsibilities within their field (Cruess et al., 2014, 2015, 2016), serves as a foundational concept for understanding IPI. Building on this, IPI can be defined as “the development of a robust cognitive, psychological, and emotional sense of belonging to an interprofessional community necessary to achieve shared context-dependent goals” (Tong et al., 2020). While this definition aligns with literature emphasizing collective belonging and context-driven collaboration, it is important to note that IPI remains an evolving construct with varying conceptualizations across studies (Cantaert et al., 2022; Tong et al., 2020). Research underscores the significance of identity formation during the undergraduate education, with Ganotice (2023) emphasizing the transition from professional identity to IPI as vital for effective teamwork and improved patient outcomes. Expanding the conceptualization of identity formation from professional to interprofessional responds to the changing roles of healthcare professionals, emphasizing the importance of interprofessional teamwork in managing complex healthcare needs (Khalili et al., 2013; McNair, 2005). Existing studies explored the development of IPI among health professions students, which can be shaped through interprofessional learning and practice (Haugland et al., 2019; He et al., 2024; Rees et al., 2019; Tong et al., 2021). In review studies, both Polansky et al. (2022) and Tong et al. (2020) concur that examining the construct of IPI is a critical agenda that warrants further investigation, as it could potentially support the necessary “paradigm shift” to transform healthcare practice towards interprofessionality (D’amour & Oandasan, 2005). Despite growing interest in IPI development, a systematic understanding of its formation is still lacking (Reinders et al., 2020). This gap underscores the need for further research efforts to comprehensively explore and elucidate the factors and paths involved in the development of IPI among health professions students.

**Fig. 1:**
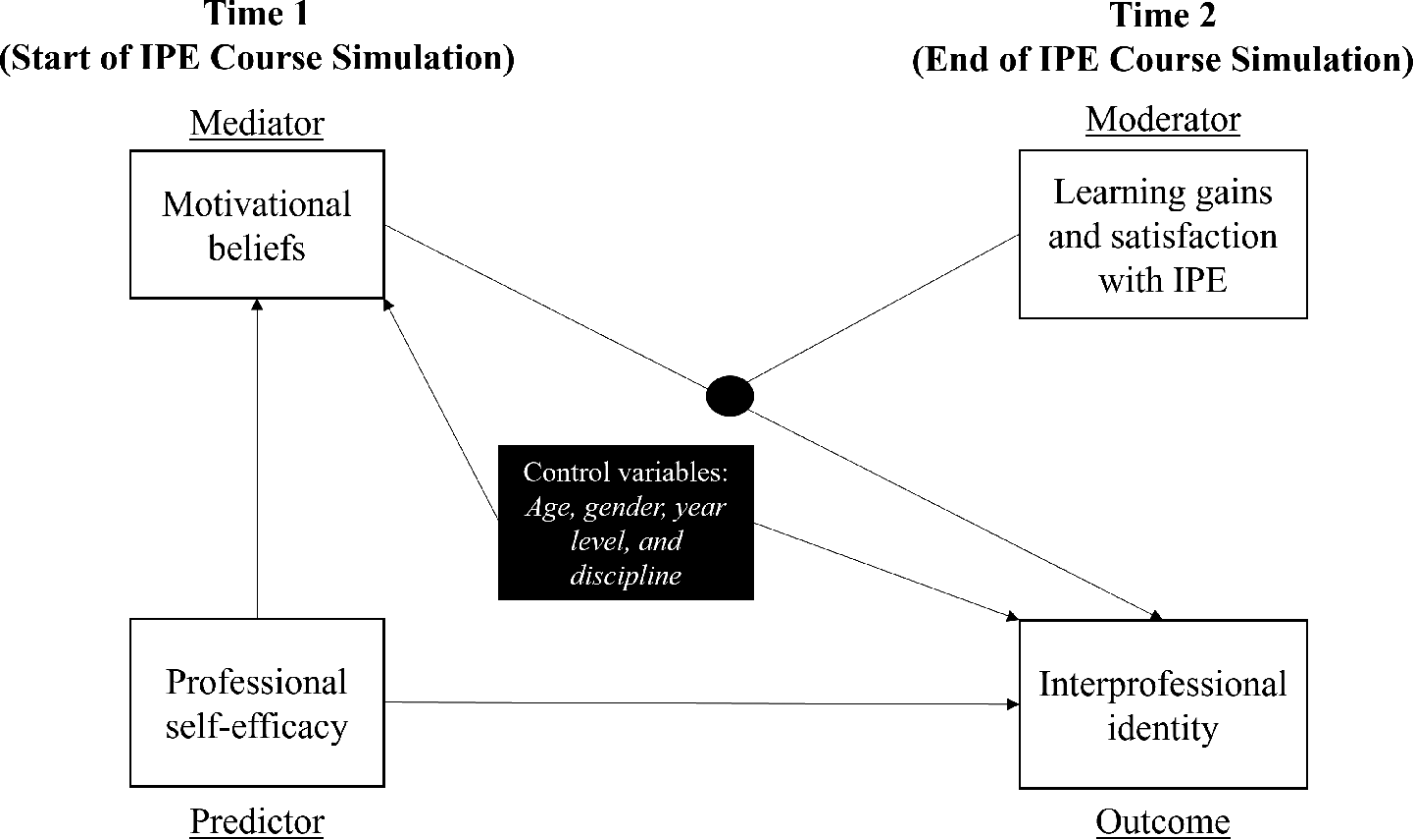
The hypothesized moderated mediation model. *Note*: This figure shows the hypothesized partial mediation of Time 1 Motivational beliefs on the link between Time 1 Professional self-efficacy and Time 2 Interprofessional identity moderated by Time 2 Learning gains and satisfaction with IPE while controlling for age, gender, year level, and discipline. All paths are hypothesized to be positive

In light of these gaps and grounded in positivist philosophy, this study proposes a theoretical model that examines the pathway from professional self-efficacy to the development of IPI, and test the mediating role of motivational beliefs and moderating of learning gains and satisfaction in IPE. We illustrate our conceptual framework in Fig. 1.

### Professional self-efficacy and IPI

A recent systematic review highlights the significance of self-efficacy in constructing IPI (Cantaert et al., 2022). The findings suggest that individual beliefs play a critical role in determining professionals’ engagement levels and their perceived capabilities when experiencing ease and confidence. Professional self-efficacy, characterized by the belief in being “confident to do”, pertains to individuals’ perceptions of their competencies and roles in their professional spheres. According to Bandura (1982), self-efficacy represents individuals’ beliefs in their ability to effectively accomplish tasks through appropriate actions. In the realm of medical education, self-efficacy beliefs significantly impact students’ dedication and persistence, positively correlating with academic performance (e.g., Tiyuri et al., 2018; Zheng et al., 2021). Notably, specific self-efficacy beliefs can predict behaviors and attitudes within certain contexts (Sherer et al., 1982). Hackett and Betz (1981) introduced the concept of professional self-efficacy, highlighting its potential to yield desired outcomes and shape perceptions of the work environment. Professional self-efficacy involves students personally identifying with the learning process to become professionals and their confidence in successfully mastering the requisite skills (Tan et al., 2017). However, there is limited evidence on how professional self-efficacy relates to IPI, a gap that this study aims to address.

### The mediating role of motivational beliefs

In addition, we propose that motivational beliefs, encompassing values and expectancies towards interprofessional learning, serve as a mediating factor between professional self-efficacy and IPI. High levels of professional self-efficacy are linked to increased motivation, resilience, and better engagement at work (Salanova et al., 2011), leading to elevated effort and persistence among individuals (Williams et al., 2017). The efficacy of teaching and learning hinges on student motivation in the classroom, with motivational strategies fostering student interest and investment in the learning process (Alcivar et al., 2020).

Drawing on the expectancy-value theory (Wigfield, 1994; Wigfield & Eccles, 2000), which elucidates the relationship between students’ expectancy of success and the value they place on task completion or goal achievement, we propose that students’ motivational beliefs, incorporating both value and expectancy components towards interprofessional learning, mediate the link between professional self-efficacy and IPI. This theoretical framework can elucidate how learners’ professional self-efficacy influences their motivational beliefs in interprofessional learning (Boström and Palm, 2020, Poort et al., 2023, Rosenzweig et al., 2019). According to this theory, learners’ motivation is shaped by their belief in success and the significance they attribute to a task. In the context of interprofessional learning, students with high professional self-efficacy are more likely to harbor positive expectations of success and value the learning process, thereby influencing their motivational beliefs. This active engagement fosters the development of a robust IPI, as students actively participate in the learning process and comprehend the importance of interprofessional collaboration.

### The moderating role of learning gains and satisfaction with IPE

The pathway from professional self-efficacy to IPI is intricate and necessitates a catalyst to facilitate the transformative journey. Interprofessional training within the curriculum, focusing on enhancing learners’ interprofessional competencies, emerges as a key precursor to IPI (Cantaert et al., 2022). Ganotice (2023) posits that both external and internal factors influence IPI, with the design of IPE programs serving as an external determinant. Reinders and Krijnen (2023) further advocate that well-structured IPE and collaborative practices can act as contextual triggers, fostering student engagement in collaborative educational settings and ultimately nurturing IPI. Polansky et al. (2022) recommend future research exploring how learning contexts impact IPI formation in the review study.

Drawing on the Kirkpatrick (1959) model, the reaction level serves as a foundational aspect for gauging learners’ perceptions of satisfaction, engagement, and relevance within educational training. Learning gains and satisfaction reflect students’ perceptions of their learning experiences (Kuo et al., 2014) and acquisition (McGrath et al., 2015). Investigating students’ perceived learning gains and satisfaction with IPE can aid educators and practitioners in optimizing educational programs and creating conducive learning environments to foster IPI development.

In this context, we propose that learners’ perceived satisfaction with the IPE curriculum acts as a moderating factor that influences their values and expectancies towards interprofessional learning, thereby shaping IPI. Positive experiences with IPE have the potential to enhance learners’ attitudes towards collaboration, thereby nurturing the development of an IPI. Satisfaction with interprofessional learning can influence professionals’ readiness to engage in collaborative practices, share knowledge across disciplines, and foster trust within interprofessional teams (Curran et al., 2008). Understanding the role of learners’ satisfaction with IPE in transitioning from perceived values and expectancies towards IPE to IPI development is crucial.

### The present study

Taken together, the present study aims to address these complex dynamics by conceptualizing a model that elucidates how students develop IPI. By exploring these intertwined factors, the study seeks to enhance our understanding of how health and social care professionals navigate professional self-efficacy, motivational beliefs in IPE, and collaborative learning experiences to foster a shared IPI. Through this examination, the study intends to provide insights that can guide interventions aimed at promoting effective interprofessional collaboration and improving the quality of care delivery. Therefore, we hypothesized that:

#### H1

Professional self-efficacy will significantly predict IPI.

#### H2

Professional self-efficacy will significantly predict motivational beliefs.

#### H3

Motivational beliefs will significantly predict IPI.

#### H4

Motivational beliefs will partially mediate the link between professional self-efficacy and IPI.

#### H5

Learning gains and satisfaction with the IPE program will moderate the link between motivational beliefs and IPI.

## Materials and methods

### Study design and procedures

This quantitative longitudinal study was conducted during a 3-week IPE simulation course implemented at a university in Hong Kong (see Supplementary Fig. 1). We collected data from consenting participants at the start (Week 1; Time 1) and end (Week 3; Time 2) simulation course in 2023. Participants were provided with an online survey link (administered through Qualtrics) before and after their participation in the simulation course. Informed consent was provided to the participants at the beginning of the survey and their consent or non-consent to voluntarily participate had no bearing in any aspect of their academic assessment for the simulation course. The study’s ethics and procedures were reviewed and approved by the Human Research Ethics Committee for Non-clinical Faculties at the University of Hong Kong, with the approval number EA210433 ensuring compliance with ethical research standards.

### Context

The IPE simulation course within which the study was conducted was designed based on the team-based (Michaelsen et al., 2002) and case-based learning (Thistlethwaite et al., 2012) pedagogies. Combining such pedagogies, the course was designed to promote the development of problem-solving, clinical reasoning, collaborative leadership, and conflict-resolution skills. The students progressed through the three-part IPE simulation course that spanned three weeks.

During Part 1 (Week 1; Preparation, Online self-directed learning), the students went through various activities that aimed to develop their team cohesiveness and identity. These included building rapport by meeting team members in a learning management system, reading the pre-class study materials (e.g., book chapters, journal articles, reports, guidelines, etc.) as a group and asking questions to each other about the reading materials, writing multiple-choice questions (MCQs) based on the pre-class study materials, and creating the team’s name. In Part 2 (Week 2; Readiness Assurance Process and Application Exercise, Interprofessional small group meeting and learning), students met their teammates in person. They answered a “readiness assurance test” individually and then as a team. The test was based on the pre-class study materials that the students had read in Part (1) When taking the test as a team, they could discuss each item and clarify the individual members’ thought processes in determining the best answer. Next, in an application exercise, the interprofessional teams learned about the patient’s case (i.e., an infectious disease case) and were tasked to design an integrated interprofessional healthcare plan. Finally, in Part 3 (Week 3; Enrichment Activity, Debriefing session), all the teams gathered in a large-group session to dialogue with the content experts (i.e., an interprofessional team of health and social care experts) about the answers to the test they had taken in Part (2) The teams received feedback from the content experts about their interprofessional healthcare plans and shared their reflections on their teamwork experience.

A subset of participants (i.e., Medicine, Nursing, and Pharmacy students) had prior interprofessional training experiences but in different clinical cases (e.g., dementia management) and varied interprofessional team compositions. We posit that recurring activities, such as collaborative readiness assurance testing and designing interprofessional care plans, provided iterative opportunities for participants to refine teamwork competencies and internalize interprofessional values. These task-based experiences align with established frameworks linking repeated, reflective collaboration to IPI development.

### Participants

Four hundred and seventy-three undergraduate health professions students participated in this study (50.53% response rate). They were from Chinese Medicine (*n* = 16; 3.38%), Medicine (*n* = 59; 12.47%), Nursing (*n* = 191; 40.39%), Social Work (*n* = 13; 2.75%), Pharmacy (*n* = 49; 10.36%), Physiotherapy (*n* = 116; 24.52%), Biomedical engineering (*n* = 11; 2.33%), and Economics (*n* = 18; 3.80%). The mean age of the participants is 21.01 (SD = 1.90), ranging from 18 to 33 years old. More than half of the participants were females (*n* = 279; 58.99%). Lastly, the participants were from different year levels of study: Year 1 (*n* = 18; 3.81%), Year 2 (*n* = 120; 23.56%), Year 3 (*n* = 166; 35.10%), Year 4 (*n* = 91; 19.24%), Year 5 (*n* = 72; 15.22%), and Year 6 (*n* = 6; 1.27%). Table 1 shows the summary of the demographic characteristics of the participants.

**Table 1:** Demographic characteristics

Characteristic	*n*	%
**Gender**		
Female	279	58.99
Male	194	41.01
**Discipline**		
Chinese Medicine	16	3.38
Medicine	59	12.47
Nursing	191	40.39
Social Work	13	2.75
Pharmacy	49	10.36
Physiotherapy	116	24.52
Engineering	11	2.33
Economics	18	3.80
**Year level**		
1	18	3.81
2	120	25.36
3	166	35.10
4	91	19.24
5	72	15.22
6	6	1.27

*Note*: *n* = 473. Participants’ mean age is 21.01 (SD = 1.90), ranging from 18 to 33 years old

### Measures

#### Demographic covariates

We collected the participants’ age, gender, year level, and discipline as demographic covariates for this study.

#### Professional self-efficacy

We used the six-item professional self-efficacy subscale of the 27-item Professional Identity Five Factor Scale (Tan et al., 2017) to measure professional self-efficacy. A sample item includes “*I’m confident that I can do an excellent job in the future*.” Participants responded to the items using a five-point Likert scale ranging from “1 = never true” to “5 = definitely true”. Higher mean scores represent greater professional self-efficacy. Participants completed this scale in Time 1 (i.e., at the start of the IPE teaching). The Cronbach’s alpha for the scale in the current study is α = 0.75.

#### Motivational beliefs

We used the 26 items from the motivation scales of the 44-item Motivated Strategies for Learning Questionnaire (MSLQ; Pintrich et al., 1993). The motivation scales include both value and expectancy components. Value components items include intrinsic goal orientation (4 items; “*In a class like this*,* I prefer study learning material that really challenges me so I can learn new things*”), extrinsic goal orientation (4 items; “*Getting a good grade in IPE is the most satisfying thing for me right now*”), task value (6 items; “*I think I will be able to use what I learn in IPE in other courses*”); and expectancy components of motivation consist of control of learning beliefs (4 items; “*If I study in appropriate ways*,* then I will be able to learn the material in IPE*”), and self-efficacy for learning and performance (8 items; “*I believe I will receive an excellent grade in IPE*”). Participants responded to the items using a Likert scale from 1 (not at all true of me) to 7 (very true of me). Participants completed this scale in Time 1. The Cronbach’s alpha for this scale in the current study is α = 0.97.

#### Interprofessional identity

We used the 12-item Extended Professional Identity Scale (EPIS; Reinders et al., 2020) to measure IPI. This scale has three dimensions: interprofessional belonging, e.g., “*I like meeting and getting to know people from other health professions*”, interprofessional commitment, e.g., “*I would be very happy to spend the rest of my career with an interprofessional team*,” and interprofessional beliefs, e.g., “*Interprofessional team members should jointly agree to communicate plans for patient care*.” Participants responded to the items using a five-point Likert scale ranging from “1 = strongly disagree” to “5 = strongly agree”. Higher mean scores on the overall scale imply greater interprofessional identity. Participants completed this scale in Time 2. The scale’s Cronbach’s alpha for the current study is α = 0.96.

#### Learning gains and satisfaction with the IPE program

We based the reaction level of Kirkpatrick’s (1959) typology to create a 13-item scale to measure the students’ perceived learning gains and satisfaction with their IPE learning experience. Five items are designed to measure students’ perceived learning gains after participating in IPE. Sample items include, “*The program was helpful for understanding the importance of teamwork*.” The other eight items are relevant to evaluating students’ satisfaction with IPE learning. Questions are related to rating program objectives, course materials, learning management platform, content relevance, etc. Sample items include, “*The purpose of the program was clearly explained to the students*.” The participants responded to the items using a Likert scale from 1 (strongly disagree) to 5 (strongly agree). Higher mean scores imply greater satisfaction with the IPE program. Participants completed this scale in Time 2. The scale’s Cronbach alpha for the current study is α = 0.97.

### Data analysis

To reiterate, we collected data at two time-points: Time 1 (at the start of the IPE simulation course) and Time 2 (at the end). Professional self-efficacy and motivational beliefs were measured at Time 1, while interprofessional identity (IPI) and learning gains and satisfaction with the IPE program were measured at Time 2. Data cleaning, descriptive statistics, Pearson’s R correlation analysis, and moderated mediation analysis via PROCESS Macro (Model 14) version 4.2 (Hayes, 2013) were performed in SPSS version 28.

Briefly, a moderated mediation model tests for a mediation model that includes an interaction between the mediator variable and a moderator variable in predicting the outcome variable (Hayes, 2018). In other words, a moderated mediation is observed when the strength of the an indirect effect depends on the level of another variable (Preacher et al., 2007).

In this study, we used three interlinked steps in conducting our moderated mediation analysis:

First, we tested a simple mediation model (PROCESS Macro Model 4; Hayes, 2017) where we regressed the outcome variable simultaneously (Time 2 IPI) to the predictor variable (Time 1 professional self-efficacy), the mediator variable (Time 1 motivational beliefs), and the demographic covariates (i.e., gender, age, year level, and discipline) in the model. We also regressed the mediator variable to the demographic covariates to further control for their potential influence within the mediation model.

Second, we ran the same simple mediation model above but we included another covariate (Time 2 learning gains and satisfaction with the IPE program) in the same model to test if the mediating role of Time 1 motivation beliefs would still hold statistical significance when the covariate was included. Given that Time 1 motivation beliefs became non-significant after including Time 2 learning gains and satisfaction with the IPE program, we further performed additional analyses in the next step.

Lastly, we tested a moderated mediation model (PROCESS Macro Model 14; Hayes, 2017), where we regressed the same outcome variable to the same predictor and mediator variables from the first model, and added the variable Time 2 learning gains and satisfaction with the IPE program as a moderator in the link between Time 1 motivational beliefs (mediator) and Time 2 IPI. See Fig. 1 for an illustration of the conceptual framework of the full moderated mediation model.

For all three models, we used the bootstrapping technique (Preacher & Hayes, 2008) with 5,000 resampled bootstraps to test mediation and moderation effects. Significant indirect effects were indicated by bias-corrected confidence intervals not containing zero.

## Results

### Descriptive statistics and bivariate correlations

The descriptive statistics and bivariate correlations are shown in Table 2. Bivariate correlation results reveal that IPI has a positive moderate relationship with professional self-efficacy (*r*(473) = 0.31, *p* < 0.001), motivational beliefs (*r*(473) = 0.31, *p* < 0.001), and learning gains and satisfaction (*r*(473) = 0.60, *p* < 0.001). Also, professional self-efficacy has a positive moderate relationship with motivational beliefs (*r*(473) = 0.42, *p* < 0.001) and learning gains and satisfaction (*r*(473) = 0.26, *p* < 0.001). Lastly, motivational beliefs also have a positive moderate relationship with learning gains and satisfaction (*r*(473) = 0.35, *p* < 0.001).

**Table 2:** Descriptive statistics, intercorrelations, and scale internal consistencies of variables (*n* = 473)

Variable	1	2	3	4
1. Interprofessional identity	(0.96)			
2. Professional self-efficacy	0.31^*^	(0.75)		
3. Motivational beliefs	0.31^*^	0.42^*^	(0.97)	
4. Learning gains and satisfaction	0.60^*^	0.26^*^	0.35^*^	(0.97)
Mean	3.81	3.46	4.47	3.69
SD	0.63	0.59	1.02	0.76
Kurtosis	0.95	0.87	0.81	1.41
Skewness	-0.43	-0.14	-0.38	-0.78

*Note*: Scores shown in parentheses on the diagonal are internal consistency reliabilities of the scales (Cronbach’s alpha). All correlations are significant. ^*^p < 0.001

### Tests of mediation

The results of testing hypotheses 1–4 are shown in Table 3. While controlling for age, gender, year level, and discipline, multiple linear regression results via PROCESS macro model 4 revealed that the positive direct effect of professional self-efficacy on IPI (Path *c’*) is significant (β = 0.224, SE = 0.053, *p* < 0.001, 95% CI [0.138, 0.346]), supporting H_1_. In addition, the positive direct effect of professional self-efficacy on motivational beliefs (Path *a*) is also significant (β = 0.420, SE = 0.074, *p* < 0.001, 95% CI [0.589, 0.881]), supporting H_2_. Further, the positive direct effect of motivational beliefs on IPI (Path *b*) is also significant (β = 0.224, SE = 0.033, *p* < 0.001, 95% CI [0.073, 0.205]), supporting H_3_. Lastly, results also revealed that the positive indirect effect of professional efficacy on IPI through motivational beliefs (Path *a* x *b*) is significant (β = 0.094, BootSE = 0.025, 95% BootCI [0.047, 0.146]). Altogether, these results suggest that motivational beliefs partially mediate the link between professional self-efficacy and IPI, supporting H_4_. The overall model accounted for 11.3% of the variance in IPI.

**Table 3:** Motivational beliefs partially mediate the link between professional self-efficacy and interprofessional identity (*n* = 473)

					Std. est.	SE	*p*	95% Confidence Intervals	
								LLCI	ULCI
*Covariates*									
Age	→	Interprofessional identity			-0.071	0.018	0.180	-0.059	0.011
Gender	→	Interprofessional identity			-0.093	0.057	0.036	-0.232	-0.008
Year level	→	Interprofessional identity			0.050	0.029	0.341	-0.030	0.086
Discipline	→	Interprofessional identity			-0.056	0.011	0.240	-0.034	0.009
Age	→	Motivational beliefs			-0.115	0.033	0.057	-0.126	0.002
Gender	→	Motivational beliefs			-0.001	0.086	0.979	-0.171	0.167
Year level	→	Motivational beliefs			-0.027	0.050	0.624	-0.122	0.073
Discipline	→	Motivational beliefs			0.106	0.016	0.015	0.008	0.071
***Direct effects***									
Professional self-efficacy	→	Interprofessional identity			0.224	0.053	0.000	0.138	0.346
Professional self-efficacy	→	Motivational beliefs			0.420	0.074	0.000	0.589	0.881
Motivation	→	Interprofessional identity			0.224	0.033	0.000	0.073	0.205
***Indirect effect***						**Bootstrapped SE**		**95% Bootstrapped CIs**	
								**LLCI**	**ULCI**
Professional self-efficacy	→	Motivational beliefs	→	Interprofessional identity	0.094	0.025	---	0.047	0.146
***Total effect***						**SE**		**95% Confidence Intervals**	
								**LLCI**	**ULCI**
Professional self-efficacy	→	Motivational beliefs	→	Interprofessional identity	0.318	0.051	0.000	0.244	0.445
*R* ^*2*^					0.113^***^				
*F*					10.114^***^				

*Notes*: Std. est. = Standardized estimates; SE =. Standard error; LLCI = lower level confidence interval; ULCI = upper level confidence interval. Bootstrapped SE and CIs were based on 5,000 bootstrap samples. A heteroscedasticity consistent standard error and covariance matrix estimator were used. ^***^ = *p* < 0.001

Given that we also inquired from the participants about their level of learning gains and satisfaction after the IPE program, we tested the same mediation model as above and added the variable learning gains and satisfaction as a covariate. Using the same mediation analysis as above, results (see Supplementary Table 1) revealed that the positive direct effects of professional self-efficacy on motivational beliefs (β = 0.356, SE = 0.076, *p* < 0.001, 95% CI [0.474, 0.773]) and IPI (β = 124, SE = 0.047, *p* = 0.004, 95% CI [0.042, 0.226]) are still significant. However, with the inclusion of learning gains and satisfaction as a covariate, the positive direct effect of motivational beliefs (β = 0.080, SE = 0.029, *p* = 0.085, 95% CI [-0.007, 0.106]) and its previous partial mediation effect were both found to be non-significant (β = 0.029, BootSE = 0.016, 95% BootCI [-0.002, 0.063]). The overall model explained around 39% of the variance in IPI (R2 = 0.39, F(3,469) = 49.94, *p* < 0.001).

### Test of moderated mediation

**Fig. 2:**
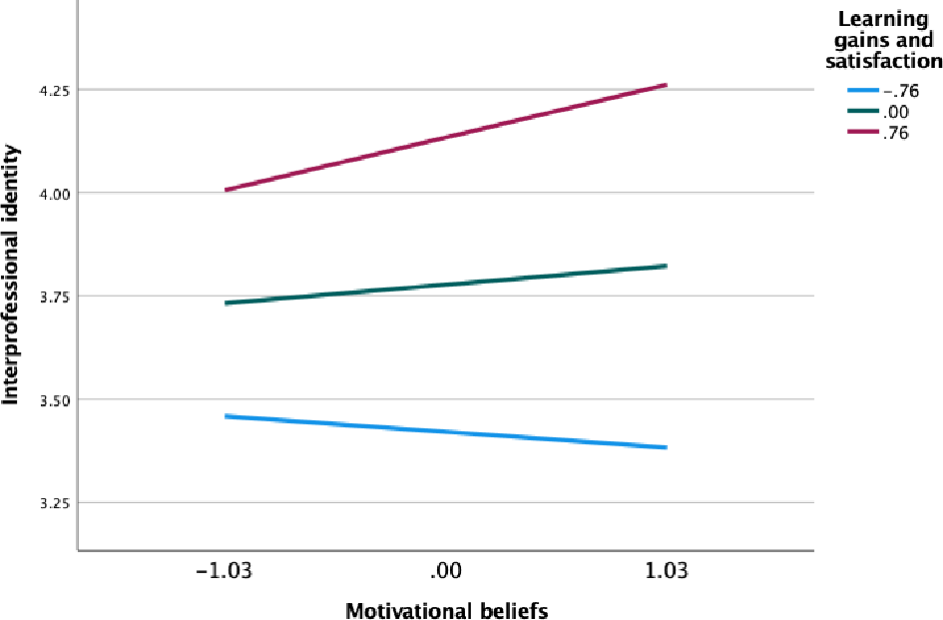
Simple slopes plot for the moderating role of learning gains and satisfaction on the link between motivational beliefs and interprofessional identity

**Fig. 3:**
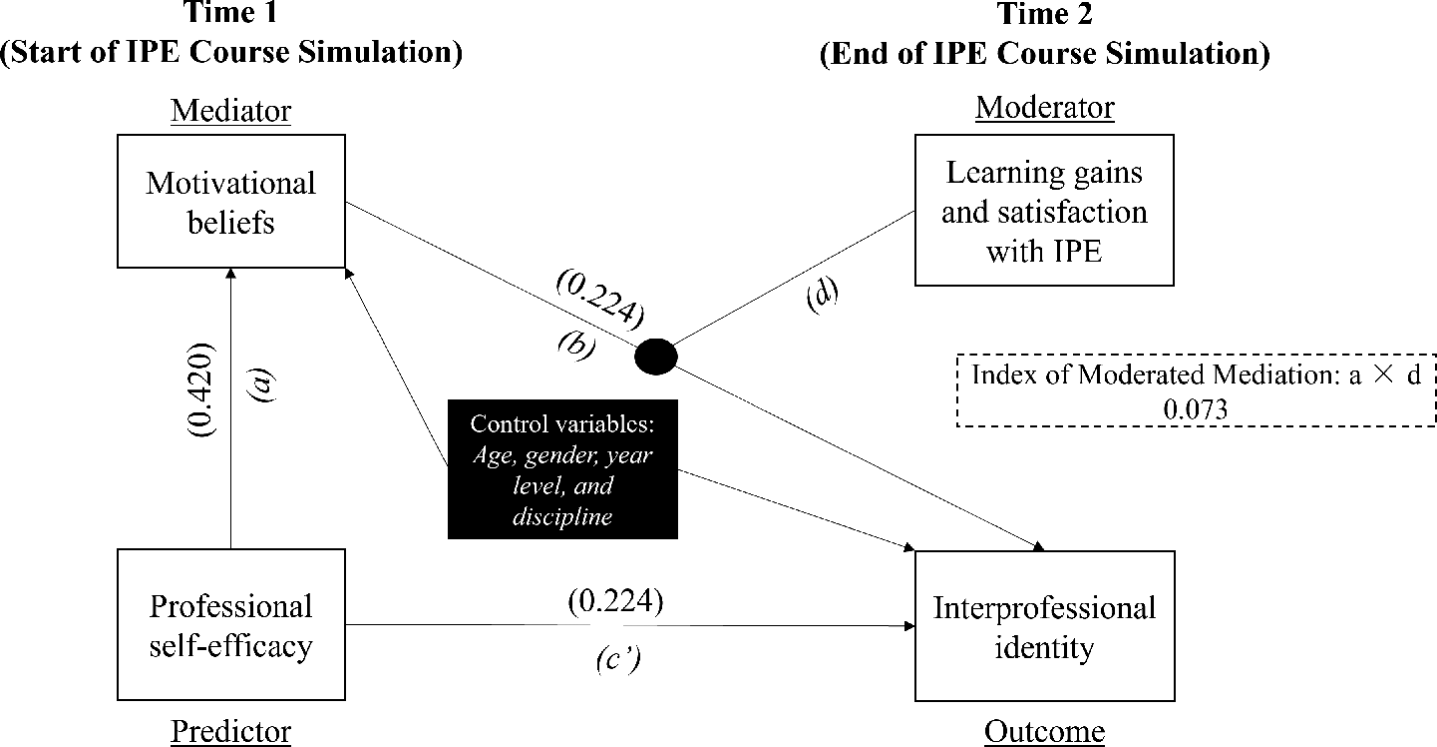
The resulting moderated mediation model. *Note*: This figure illustrates the partial mediating role of Time 1 motivational beliefs on the link between Time 1 professional self-efficacy and Time 2 interprofessional identity moderated by Time 2 learning gains and satisfaction with IPE. All paths are positive and were significant at *p* < 0.001

Following the non-significant mediation results after including the covariate learning gains and satisfaction, we further probed for a potential moderated mediation model where we hypothesized that learning gains and satisfaction will moderate the indirect effect of professional self-efficacy on IPI through motivational beliefs (H5). While still controlling for the same demographic covariates in the previous mediation models, regression-based conditional process analysis via PROCESS macro model 14 results (see Table 4) reveal that the interaction effect of motivational beliefs × learning gains and satisfaction on IPI is significant (*B* = 0.099, SE = 0.039, *p* < 0.05, 95% CI [0.022, 0.120]). Further, the conditional indirect effect of professional efficacy on IPI through motivational beliefs was significant only at both high (*B* = 0.096, BootSE = 0.028, 95% BootCI [0.043, 0.155]) and average (*B* = 0.040, BootSE = 0.022, 95% BootCI [0.001, 0.086]) learning gains and satisfaction. We illustrate this result through a simple slopes analysis plot (see Fig. 2). Lastly, the index of moderated mediation is also significant (B = 0.073, BootSE = 0.026, 95% BootCI [0.020, 0.120]). The final overall model (see Fig. 3) accounted for around 41% of the variance in IPI (R2 = 0.41, F(4,468) = 43.12, *p* < 0.001).

**Table 4:** The moderating effect of learning gains and satisfaction on the mediating role of motivational beliefs in the link between professional self-efficacy and interprofessional identity (*n* = 473)

					Un.Est.	SE	*p*	95% Confidence Intervals	
								LLCI	ULCI
*Covariates*									
Age	→	Interprofessional identity			-0.013	0.017	0.473	-0.047	0.022
Gender	→	Interprofessional identity			-0.027	0.046	0.565	-0.117	0.064
Year level	→	Interprofessional identity			0.071	0.025	0.005	0.021	0.120
Discipline	→	Interprofessional identity			-0.023	0.009	0.015	-0.041	-0.004
Age	→	Motivational beliefs			-0.062	0.033	0.057	-0.126	0.002
Gender	→	Motivational beliefs			-0.002	0.086	0.979	-0.171	0.167
Year level	→	Motivational beliefs			-0.024	0.050	0.624	-0.122	0.073
Discipline	→	Motivational beliefs			0.040	0.016	0.015	0.008	0.071
***Direct effects***									
Professional self-efficacy	→	Interprofessional identity			0.107	0.046	0.020	0.017	0.197
Professional self-efficacy	→	Motivational beliefs			0.735	0.074	0.000	0.589	0.881
Motivational beliefs	→	Interprofessional identity			0.055	0.030	0.070	-0.005	0.115
Learning gains and satisfaction	→	Interprofessional identity			0.496	0.050	0.000	0.399	0.593
***Interaction effect***									
Motivational beliefs × Learning gains and satisfaction	→	Interprofessional identity			0.099	0.039	0.012	0.022	0.120
***Conditional indirect effects***						**Bootstrapped SE**		**95% Bootstrapped CIs**	
								**LLCI**	**ULCI**
Low learning gains and satisfaction (-1SD)					-0.015	0.030	----	-0.069	0.049
Average learning gains and satisfaction (at the mean)					0.040	0.022	----	0.001	0.086
High learning gains and satisfaction (+ 1SD)					0.096	0.028	----	0.043	0.155
*Index of moderated mediation*					0.073	0.026	----	0.020	0.120
*R* ^*2*^					0.445^***^				
*F*					41.004^***^				

*Notes*: Un.Est. = Unstandardized estimates; SE =. Standard error; LLCI = lower level confidence interval; ULCI = upper level confidence interval. Bootstrapped SE and CIs were based on 5,000 bootstrap samples. A heteroscedasticity consistent standard error and covariance matrix estimator were used. ^***^ = *p* < 0.001

## Discussion

This study supported our hypotheses: students who reported high professional self-efficacy were more likely to develop their sense of IPI (H1). Additionally, motivational beliefs mediated the relationship between professional self-efficacy and IPI (H4), indicating that students who perceived higher professional self-efficacy tend to have higher motivational beliefs towards interprofessional learning (H2), which in turn helps them to develop higher-level IPI (H3). Students perceived learning gains and satisfaction with IPE moderated the association between motivational beliefs and IPI development (H5).

### Professional self-efficacy and interprofessional identity

Based on our results, there was a direct effect of professional self-efficacy on IPI. This indicates that an individual’s confidence in their professional skills directly boosts their sense of inclusion and connection within an interprofessional team. When individuals are assured of their competencies, they are more willing and capable of collaborating with other professionals. This assurance can alleviate anxiety and defensiveness in interprofessional settings, fostering improved collaboration and integration of diverse professional viewpoints (Cahn et al., 2018). Conversely, students with lower professional self-efficacy may encounter challenges navigating the intricate dynamics of interprofessional teamwork, potentially leading to role ambiguity or conflict (Nurumal et al., 2020).

In our study, students from various disciplines were organized into interprofessional teams. We emphasized collaborative practices throughout the learning activities, involving communication in intergroup scenarios, like collaborations in interprofessional teams. These activities encompassed both intragroup and intergroup communication among medical care, social care, and other relevant disciplines. A critical distinction between intergroup and intragroup communication lies in individuals’ awareness of their group affiliations. Those perceiving their identities as multifaceted may exhibit unique communication patterns within interprofessional teamwork, potentially displaying behaviors that reduce social barriers and enhance acceptance and trust levels (Monrouxe, 2010).

According to social identity theory, individuals tend to exhibit behaviors that align with the interests of their social group (Tajfel, 1982). Consequently, those who identify strongly with a group are inclined to engage in behaviors that advance the group’s interests and contribute to collective goals (Abrams & Hogg, 1988; Ellemers et al., 1998, 1999; Tyler, 1999; Tyler & Blader, 2003). Self-efficacy is considered a crucial element in understanding the link between social identity and behaviors that support group interests (Guan & So, 2016). Given self-efficacy’s potential impact on future work performance, resilience, and motivation (Ommering et al., 2021), it is crucial for students to cultivate confidence in their ability to perform effectively upon graduation. Ideally, students should view themselves as developing professionals capable of sound judgment and adeptly handling diverse professional scenarios through the application of pertinent knowledge, skills, tools, and resources. Research by Xiong et al. (2020) indicates that self-efficacy directly influences learners’ professional identity. Conceptually, professionals’ perceptions of their identities have been found to affect interactions within interprofessional teams (Fitzgerald & Teal, 2004). Arguably, our study extends this understanding to an interprofessional context, reinforcing the concept of transforming professional self-efficacy into IPI.

### Motivational beliefs: bridging the concept between professional self-efficacy and interprofessional identity

Our results also reveal that motivational beliefs toward interprofessional learning approached from value and expectancy perspectives, serve as a mediator between professional self-efficacy and IPI. This finding not only aligns with previous findings but also expands upon them by emphasizing the pivotal role of constructivist learning environments in fostering IPI development, which can be significantly influenced by motivational factors (Cantaert et al., 2022).

Doménech-Betoret et al. (2017) demonstrated that expectancy-value beliefs mediate the relationship between self-efficacy and academic achievement. Reinders and Krijnen (2023) explored the correlation between IPI and motivation toward interprofessional collaboration, hinting at a potential interlinkage between these constructs. In our current investigation, value beliefs and expectancy reflect how students perceive their worth and their confidence in engaging in interprofessional learning. Individuals with high professional self-efficacy who acknowledge the relevance and advantages of interprofessional collaboration are more inclined to actively participate in interprofessional learning endeavors with assurance in their ability to succeed. Positive value and expectancy beliefs prompt individuals to actively seek interprofessional experiences, dedicate time and effort to acquire interprofessional competencies, engage actively, demonstrate readiness to tackle interprofessional challenges, and exhibit persistence in overcoming obstacles. As professionals immerse themselves in interprofessional learning and witness the affirmative outcomes of collaboration, their sense of IPI deepens.

Moreover, the formation of IPI is shaped by fundamental beliefs indicative of an interprofessional orientation, underscoring the significance of IPI-aligned constructivist learning environments and intergroup leadership in its cultivation (Cantaert et al., 2022). Exposure to IPE transforms facets of students’ professional identity construction, influencing their sense of belonging and ability to identify with both their individual discipline and the broader interprofessional community (Bloomfield et al., 2020). IPI embodies an acknowledgment of the value of collaboration, comprehension of one’s role within the interprofessional team, and a dedication to collaborative efforts aimed at enhancing patient care and outcomes. To nurture the development of IPI, educational programs, and professional development initiatives should prioritize the cultivation of value and expectancy beliefs. By emphasizing the significance of interprofessional collaboration and providing avenues for active learning, skill enhancement, and reflective practices, professionals can bolster their value beliefs and reinforce their commitment to interprofessional practice. Additionally, offering structured learning experiences and feedback can elevate health professions students’ expectancy beliefs, boosting their confidence in their interprofessional competencies and fortifying their sense of IPI.

### The role of learners’ learning gains and satisfaction

The moderated mediation analysis reveals that the variable learning gains and satisfaction with the program significantly moderates the indirect effect of professional self-efficacy on IPI through IPI is amplified when students perceive learning gains from and are more satisfied with the program. Notably, when the variable learning gains and satisfaction with the program are included as a covariate, it appears to account for some of the variance previously explained by motivational beliefs. This suggests that learners’ perceived learning gains and satisfaction may wield considerable influence in shaping IPI, potentially overshadowing the initial impact of motivation. This underscores the importance of ensuring student satisfaction with IPE programs to foster the development of IPI. Moreover, the conditional indirect effects analysis indicated that this mediating pathway from motivational beliefs to IPI development is significant only at high and average levels of learning gains and satisfaction, but not at low levels. This finding highlights that learner learning gains and satisfaction are not merely a by-product of the educational process but a critical component that can transform students’ initial value and competency components of motivation to IPI formation.

This finding resonates with existing literature underscoring the pivotal role of student satisfaction in shaping educational outcomes (Cant et al., 2023). Furthermore, our study highlights the multifaceted influence of various factors on student satisfaction, including faculty teaching strategies, the learning environment, course content and structure, and social interactions among students and faculty (Cant et al., 2023). In addition, a review study has indicated the reciprocal nature of student satisfaction and motivational beliefs (Walker et al., 2016). Motivational beliefs, encompassing personal commitment, goal-setting, self-motivation, and support, are instrumental in driving students to persist in their learning endeavors. Building upon this foundation, our study explores deeper into this relationship by illustrating how learning gains and satisfaction can amplify the positive effects of professional self-efficacy and motivation on IPI development in the context of IPE.

To maximize the impact of IPE programs, educators and program designers should prioritize strategies that enhance learners’ learning experiences. It is essential to consider the drivers of the students’ perceptions in both cognitive (learning gains) and emotional (satisfaction) realms when aiming to foster IPI through interprofessional learning. Specifically, to consider how much they enjoyed the learning process (affective reactions) and how much they believe they have learned (utility judgments). This could involve providing supportive learning environments, ensuring the curriculum’s relevance and applicability, and nurturing positive interprofessional interactions.

In the current IPE program design, deliberate adoption of case-based learning and small group settings with peer teaching has been implemented to stimulate students’ engagement in learning. Similarly, Curran et al. (2008) observed variations in student satisfaction with IPE across disciplines, with face-to-face, case-based learning being favored across disciplines. Kooloos et al. (2011) also found that students prefer small-group learning with peer teaching. By incorporating these approaches, educators can better facilitate the development of a robust IPI among healthcare professionals, ultimately leading to enhanced collaborative practice and improved patient care outcomes.

### Strengths and limitations

This study contributes insights into the development of IPI through IPE, an area that falls short of empirical grounding in existing literature (Reinders et al., 2020). In addition to highlighting the roles of learners’ professional self-efficacy and motivational beliefs, the study underscores the significance of learning gains and satisfaction with IPE in shaping educators’ perspectives on integrating IPE into curricula and enhancing program design.

However, this study is not without its limitations. Firstly, the assessment of participants’ perceived learning gains relied on self-reported measures, which might not fully capture the extent of their educational advancements. Future research could incorporate objective tests to evaluate learners’ knowledge and skills more comprehensively. Secondly, while data were collected from participants across eight disciplines, the uneven distribution of participants (largely due to logistical and availability challenges) among these disciplines could introduce bias into the findings. Future studies could address this by recruiting a more balanced representation of participants across disciplines to compare the nuances in developing IPI. Third, although this study utilized a rigorous quantitative research design, the subjective insights of the learners regarding their interprofessional identity may have not been fully captured by this design. Future studies may consider utilizing a qualitative or a mixed-method design to gain further data on the subjective insights of the learners regarding their interprofessional identity. Lastly, while our model contributed to uncovering the pathways to IPI development, it is important to also acknowledge its limitations. As highlighted by previous studies, some learners struggle with “fitting in” professionally due to value conflicts or competing identities (Cornett et al., 2023). These identity tensions could explain variations in our findings, particularly among students with lower professional self-efficacy despite strong motivational beliefs. Future research should examine how identity struggles influence the pathway from professional self-efficacy to IPI and whether addressing these tensions might enhance IPI development. This perspective acknowledges the complex nature of professional identity formation in healthcare education.

## Conclusions

This study presents a model that empirically investigates the cultivation of IPI in students participating in IPE. Our findings suggest two key contributions to the literature. First, while professional self-efficacy appears to directly contribute to the enhancement of students’ IPI, we found that motivational beliefs, encompassing aspects of value and expectancy, may mediate this relationship. This finding implies that students with heightened professional self-efficacy might be more inclined to value and anticipate success in interprofessional learning, which could in turn reinforce their IPI. Second, learners’ perceptions of their learning progress and satisfaction with the IPE program seem to play pivotal roles in shaping how motivational beliefs connect to the formation of their IPI.

Collectively, these findings underscore the potential of educational strategies that integrate both self-efficacy development and motivational engagement to foster IPI. This study serves as an empirical foundation for advancing IPE approaches that prioritize deep, meaningful learning experiences, suggesting educators could optimize programs by designing supportive environments and collaborative activities that explicitly address learners’ values, expectations, and sense of achievement. By nurturing professional self-efficacy, motivational investment, and learner satisfaction, such initiatives may contribute to robust IPI development, which could, in turn, support enhanced teamwork and improved healthcare delivery. However, while the identified linkages between these constructs are promising, further research is needed to validate these relationships across diverse contexts, explore their long-term implications for collaborative practice, and confirm potential impacts on patient outcomes. Notably, as our conclusions are based on self-reported data, future work incorporating objective measures of IPI and longitudinal tracking of measured outcomes would strengthen these insights.

## Electronic supplementary material

Below is the link to the electronic supplementary material.

Supplementary Material 1

Supplementary Material 2

## Data Availability

The data that support the findings of this study can be requested from the corresponding author upon reasonable request.

## Abbreviations

IPE: Interprofessional education
IPI: Interprofessional identity
