# Supplementary figures and images for: Exploring pathways to develop interprofessional identity: a moderated mediation study
Source: Adv Health Sci Educ Theory Pract. 2025 May 15;31(1):145–65. doi: 10.1007/s10459-025-10441-8 (PMC12929267; doi:10.1007/s10459-025-10441-8)

**Supplementary Figure 1**

**Supplementary Figure 1**. IPE learning activities


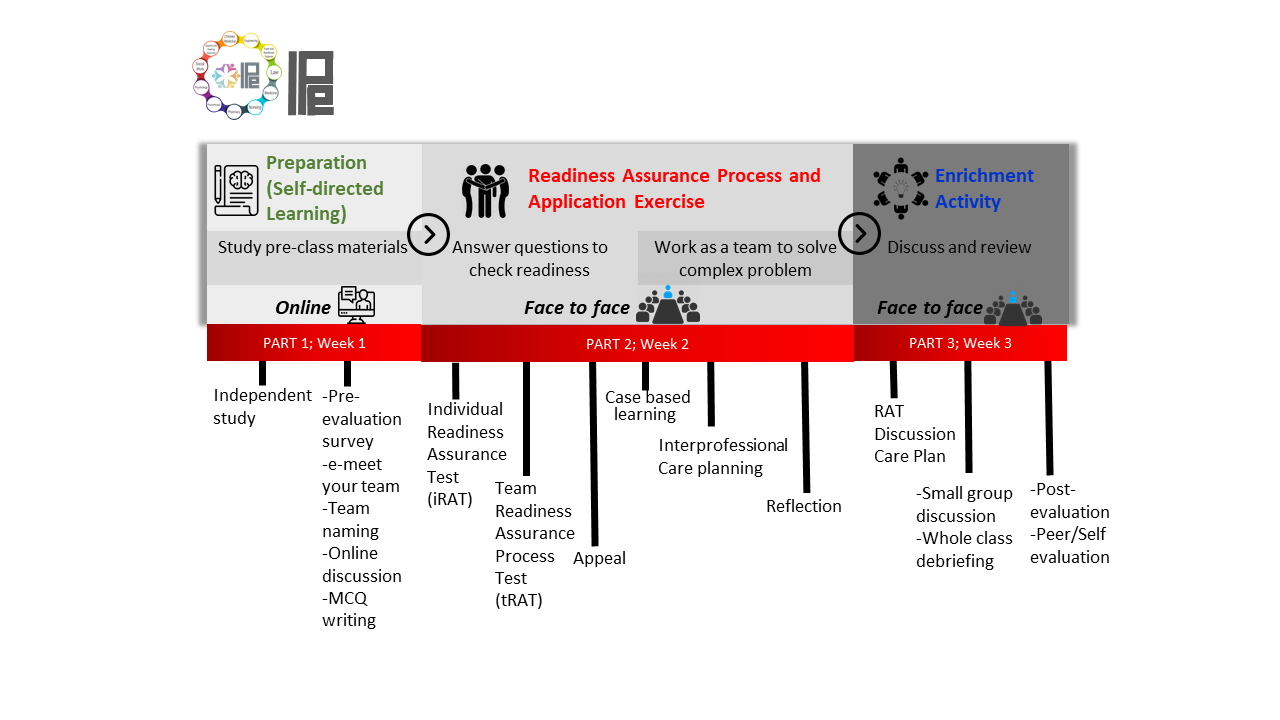

Supplement: Supplementary file 1 — Supplementary Material 1 [file 10459_2025_10441_MOESM1_ESM.docx]
