# Supplementary material for: Exploring pathways to develop interprofessional identity: a moderated mediation study
Source: Adv Health Sci Educ Theory Pract. 2025 May 15;31(1):145–65. doi: 10.1007/s10459-025-10441-8 (PMC12929267; doi:10.1007/s10459-025-10441-8)
Supplement: Supplementary file 2 — Supplementary Material 2 [file 10459_2025_10441_MOESM2_ESM.docx]

**Supplementary Table**

**Supplementary Table 1**. Mediation model with motivational beliefs mediating the link between professional self-efficacy and interprofessional identity, with learning gains and satisfaction as a covariate

|  | | | | | | | | | | | | | | | | | **95% Confidence Intervals** | | | | | | |  |  |  |
| --- | --- | --- | --- | --- | --- | --- | --- | --- | --- | --- | --- | --- | --- | --- | --- | --- | --- | --- | --- | --- | --- | --- | --- | --- | --- | --- |
|  | |  | |  | |  | |  | | | **Std. est.** | | **SE** | | ***p*** | | | | **LLCI** | | | **ULCI** | | | |  |
| *Covariates* |  |  |  |  |  |  |  |  |  |  | |  |  |  | |  | |  | |  |  | |  | |  | |
| Age |  | → |  | Interprofessional identity | | | | |  | -0.025 | |  | 0.019 |  | | 0.657 | |  | | -0.045 |  | | 0.028 | |  | |
| Gender |  | → |  | Interprofessional identity | | | | |  | -0.020 | |  | 0.047 |  | | 0.590 | |  | | -0.118 |  | | 0.067 | |  | |
| Year level |  | → |  | Interprofessional identity | | | | |  | 0.123 | |  | 0.026 |  | | 0.008 | |  | | 0.018 |  | | 0.119 | |  | |
| Discipline |  | → |  | Interprofessional identity | | | | |  | -0.110 | |  | 0.009 |  | | 0.005 | |  | | -0.043 |  | | -0.008 | |  | |
| Learning gains and satisfaction |  | → |  | Interprofessional identity | | | | |  | 0.582 | |  | 0.051 |  | | 0.000 | |  | | 0.386 |  | | 0.585 | |  | |
| Age |  | → |  | Motivational beliefs | | | | |  | -0.090 | |  | 0.030 |  | | 0.104 | |  | | -0.108 |  | | 0.010 | |  | |
| Gender |  | → |  | Motivational beliefs | | | | |  | 0.028 | |  | 0.083 |  | | 0.480 | |  | | -0.104 |  | | 0.222 | |  | |
| Year level |  | → |  | Motivational beliefs | | | | |  | 0.003 | |  | 0.050 |  | | 0.950 | |  | | -0.095 |  | | 0.102 | |  | |
| Discipline |  | → |  | Motivational beliefs | | | | |  | 0.078 | |  | 0.015 |  | | 0.055 | |  | | -0.001 |  | | 0.059 | |  | |
| Learning gains and satisfaction |  | → |  | Motivational beliefs | | | | |  | 0.231 | |  | 0.071 |  | | 0.000 | |  | | 0.171 |  | | 0.450 | |  | |
|  |  |  |  |  |  |  |  |  |  |  | |  |  |  | |  | |  | |  |  | |  | |  | |
| *Direct effects* |  |  |  |  |  |  |  |  |  |  | |  |  |  | |  | |  | |  |  | |  | |  | |
| Professional self-efficacy |  | → |  | Interprofessional identity | | | | |  | 0.124 | |  | 0.047 |  | | 0.004 | |  | | 0.042 |  | | 0.226 | |  | |
| Professional self-efficacy |  | → |  | Motivational beliefs | | | | |  | 0.356 | |  | 0.076 |  | | 0.000 | |  | | 0.474 |  | | 0.773 | |  | |
| Motivational beliefs |  | → |  | Interprofessional identity | | | | |  | 0.080 | |  | 0.029 |  | | 0.085 | |  | | -0.007 |  | | 0.106 | |  | |
| *Indirect effect* |  |  |  |  |  |  |  |  |  |  | |  | **Bootstrapped SE** |  | |  | |  | | **95% Bootstrapped CIs** | | | | |  | |
|  |  |  |  |  |  |  |  |  |  |  | |  |  |  | |  | |  | | **LLCI** |  | | **ULCI** | |  | |
| Professional self-efficacy |  | → |  | Motivational beliefs |  | → |  | Interprofessional identity |  | 0.029 | |  | 0.016 |  | | --- | |  | | -0.002 |  | | 0.063 | |  | |
| *Total effect* |  |  |  |  |  |  |  |  |  |  | |  | **SE** |  | |  | |  | | **95% Confidence Intervals** | | | | |  | |
|  |  |  |  |  |  |  |  |  |  |  | |  |  |  | |  | |  | | **LLCI** |  | | **ULCI** | |  | |
| Professional self-efficacy |  | → |  | Motivational beliefs |  | → |  | Interprofessional identity |  | 0.153 | |  | 0.045 |  | | 0.000 | |  | | 0.077 |  | | 0.254 | |  | |
| *R^2^* |  |  |  |  |  |  |  |  |  | 0.419^***^ | | | | | | | | | | | | | | |  | |
| *F* |  |  |  |  |  |  |  |  |  | 41.779^***^ | | | | | | | | | | | | | | |  | |

*Notes*: Std. est. = Standardized estimates; SE =. Standard error; LLCI = lower level confidence interval; ULCI = upper level confidence interval. Bootstrapped SE and CIs were based on 5,000 bootstrap samples. A heteroscedasticity consistent standard error and covariance matrix estimator were used. ^***^ = *p* < 0.001.
